# Diagnostic accuracy of antigen-based immunochromatographic rapid diagnostic tests for the detection of *Salmonella* in blood culture broth

**DOI:** 10.1371/journal.pone.0194024

**Published:** 2018-03-08

**Authors:** Laura M. F. Kuijpers, Panha Chung, Marjan Peeters, Marie-France Phoba, Chun Kham, Barbara Barbé, Octavie Lunguya, Jan Jacobs

**Affiliations:** 1 Institute of Tropical Medicine, Department of Clinical Sciences, Antwerp, Belgium; 2 KU Leuven, Department of Microbiology & Immunology, Leuven, Belgium; 3 Sihanouk Hospital Center of HOPE, Phnom Penh, Cambodia; 4 National Institute for Biomedical Research, Department of Clinical Microbiology, Kinshasa, the Democratic Republic of the Congo; Panjab University, INDIA

## Abstract

**Background:**

In low resource settings, *Salmonella* serovars frequently cause bloodstream infections. This study investigated the diagnostic performance of immunochromatographic rapid diagnostic tests (RDTs), which detect *Salmonella* antigens, when applied to stored grown blood culture broth.

**Material/Methods:**

The SD Bioline One Step *Salmonella* Typhi Ag Rapid Detection Kit (Standard Diagnostics, Republic of Korea), marketed for the detection of *Salmonella enterica* serovar Typhi (*Salmonella* Typhi) in stool and the *Salmonella* Ag Rapid Test (Creative Diagnostics, USA), marketed for the detection of all *Salmonella* serotypes in stool, were selected for evaluation based on a pre-test evaluation of six RDT products. The limits of detection (LOD) for culture suspensions were established and the selected RDT products were assessed on 19 freshly grown spiked blood culture broth samples and 413 stored clinical blood culture broth samples, collected in Cambodia and the Democratic Republic of the Congo.

**Results:**

The LOD of both products was established as 10^7^−10^8^ CFU/ml. When applied to clinical blood culture broth samples, the diagnostic sensitivity and specificity of the SD Bioline RDT were respectively 100% and 79.7% for the detection of *Salmonella* Typhi; 94.4% (65/69) of false-positive results were caused by *Salmonella* Enteritidis. When considering the combined detection of *Salmonella* Typhi and Enteritidis (both group D *Salmonella*), sensitivity and specificity were 97.9% and 98.5% respectively. For Creative Diagnostics, diagnostic sensitivity was 78.3% and specificity 91.0% for all *Salmonella* serotypes combined; 88.3% (53/60) of false negative results were caused by *Salmonella* Paratyphi A.

**Conclusions:**

When applied to grown blood culture broths, the SD Bioline RDT had a good sensitivity and specificity for the detection of *Salmonella* Typhi and *Salmonella* Enteritidis. The Creative Diagnostics product had a moderate sensitivity and acceptable specificity for the detection of all *Salmonella* serovars combined and needs further optimization. A RDT that reliably detects *Salmonella* Paratyphi A is needed.

## Introduction

In low and middle income countries, invasive *Salmonella* infections continue to pose an important public health problem. Enteric fever, *i*.*e*. a bloodstream infection with *Salmonella enterica* serovar Typhi (*Salmonella* Typhi) or serovar Paratyphi A, B and C (*Salmonella* Paratyphi), has an estimated incidence of 27 million cases and causes 190,200 (uncertainty range 23,800–359,100) deaths per year [1, 2]. Non-typhoidal *Salmonella* serovars, in particular *Salmonella enterica* serovar Enteritidis (*Salmonella* Enteritidis) and *Salmonella enterica* serovar Typhimurium (*Salmonella* Typhimurium) cause another 3.4 million cases of bloodstream infections and an estimated 681,316 (uncertainty range 415,164–1,301,520) deaths annually, mainly in sub-Saharan Africa [3].

Symptoms associated with these invasive infections are non-specific and laboratory diagnosis remains challenging. Blood culture is often used as the reference method but it shows poor sensitivity (40–80%) and is technically and financially demanding [4, 5]. In addition, the time to diagnosis is 2–3 days.

Other diagnostic tests like the serological Widal test or PCR-based tests suffer from low sensitivity and delayed positivity or are of little use in resource constrained settings [6]. Furthermore, incorrect use and interpretation of serological tests frequently leads to overdiagnosis and overtreatment [7].

A rapid diagnostic test that is affordable and reliable is thus urgently needed and recently developed rapid diagnostics tests (RDTs) based on *Salmonella* antigen detection seem promising. These RDTs are currently only marketed for application on stool, serum, plasma and/or whole blood and not for application on grown blood culture broth. Castonguay-Vanier *et al*. successfully applied one of them to blood culture broth speeding up the time to diagnosis of *Salmonella* Typhi infections with one to two days [8]. However, they only included samples from a single center in Laos and did not assess the detection of other frequent *Salmonella* serovars or possible cross-reactivity with Gram-positive organisms.

The present study aims to evaluate the performance of antigen-based immunochromatographic rapid diagnostic tests for the detection of the four most common *Salmonella* serovars when applied to blood culture broth from patients from Cambodia and the Democratic Republic of Congo (DRC).

## Materials and methods

### Study design

The study assessed six RDT products in three steps: (i) a proof-of-concept evaluation with determination of limit of detection (LOD); (ii) evaluation on spiked blood culture broth samples and (iii) evaluation on stored grown blood culture broth samples obtained during routine patient care in Cambodia and the DRC. The reference method was standard microbiological work-up of blood cultures; serological testing was not performed at both study sites. Reporting of the methods and results was done according to the STARD guidelines for diagnostic studies [9].

### Rapid diagnostic test products

All six RDT products assessed, their targets and abbreviations as used throughout the text are listed in Table 1. Except for the SMI Paratyphi A RDT and the SD Bioline RDT, all products are commercially available. The products are not officially marketed for application on broth. The present study is a validation study to confirm that these products can be reliably used on grown blood culture broth and can therefore speed up the time to diagnosis of invasive *Salmonella* infections; it does not concern a prospective clinical validation of these products.

**Table 1:** Characteristics of the *Salmonella* antigen-based immunochromatographic rapid diagnostic tests evaluated.

Abbreviation used in main text	Product name	Manufacturer	Specimen	No. of bands	Antigens targeted according to Instructions For Use (IFU)
**SD Bioline RDT**	SD Bioline One Step *Salmonella* Typhi Ag Rapid Detection Kit	Standard Diagnostics (SD), Republic of Korea	Stool	2	1. Monoclonal *Salmonella* Typhi antigen^a^
**Dialab RDT**	"DIAQUICK" S. typhi/paratyphi Ag cassette [10]	DIALAB GmbH, Austria	Whole blood, serum, plasma	3	1. *Salmonella* O (somatic) antigen, group D 2. *Salmonella* O antigen, group A
**Labcare RDT**	Accucare *S*. Typhi-*S*. Paratyphi Direct Antigen Detection kit [11]	Labcare, India	Serum	3	1. *Salmonella* Typhi lipopolysaccharide (LPS) antigen 2. *Salmonella* Paratyphi A LPS antigen
**SMI Typhi RDT**	*Salmonella* Typhi Antigen Strip [12]	Science with a Mission, Inc (SMI), USA	Serum, plasma	2	1. *Salmonella* Typhi antigen
**SMI (Para)typhi RDT**	*Salmonella* typhi/paratyphi A, B & C Test Device	Science with a Mission, Inc (SMI), USA	Serum, plasma	3	1. *Salmonella* Typhi antigen 2. *Salmonella* Paratyphi A antigen
**Creative Diagnostics RDT**	*Salmonella* Ag Rapid Test [13]	Creative Diagnostics (CD), USA	Stool	2	1. *Salmonella* spp. antigen

Ag = Antigen; RDT = Rapid Diagnostic Test

^a^ = Although the IFU states that the target of the test is ‘monoclonal *Salmonella* Typhi antigen’, the results as published by Castonguay *et al*. [8], suggest that the test targets the O9 antigen. This antigen can be found in all *Salmonella* serovars of serogroup D.

Included were five cassettes and one dipstick format (the SMI Typhi RDT). Per product at least two lot numbers were included. All RDT kits had been stored in compliance with the temperature stability mentioned on the box. Results were read at the minimum and maximum reading time of the reading interval described in the Instructions For Use (IFU). A test result was considered invalid when the control line was not visible. Test lines intensity was scored as ‘faint’ (hardly visible) and ‘weak’, ‘medium’ or ‘strong’ if intensity was weaker, equal or stronger compared to the control line.

### I Proof-of-concept evaluation with determination of limit of detection

*Salmonella* Typhi 00032304 (strain confirmed at the Belgian Reference Laboratory for *Salmonella*, Scientific Institute of Health (WIV-ISP), Etterbeek, Belgium), *Salmonella* Paratyphi A ATCC 9150, *Salmonella* Enteritidis ATCC 13076 and S*almonella* Typhimurium ATCC 14028 were grown overnight. Two suspensions of pure culture with a concentration of 1.5 x 10^8^ colony forming units/ml (CFU/ml) were made, one in sterile Phosphate Buffered Saline (PBS) and one in blood culture broth. Then, serial 10-fold dilutions where made to obtain a range of 1.5 x 10^8^ CFU/ml to 1.5 x 10^3^ CFU/ml. Blood culture broth was obtained from blood culture bottles (BacT/ALERT FA/FAN–bioMérieux, Marcy L’Etoile, France) which had been inoculated with 10 ml fresh whole blood from a healthy person. The accuracy of the dilutions was verified by colony count. The volumes applied to the RDTs were in compliance with the IFU of each product. In case number of drops instead of exact volumes were mentioned, exact volumes were calculated by weighing the drops applied by the kit’s transfer device. All samples were tested in duplicate.

As blood culture broth was not listed as a possible specimen for any of the RDT products tested, the sample preparation procedure was optimized. The following sample preparation steps were tested: (i) pre-incubation at 100°C for 5 minutes (PBS); (ii) centrifugation for 1 minute at 500 x *g* (blood culture broth) [8]; and (iii) dilution in buffer included in the test kits (blood culture broth and PBS).

### II Application on spiked blood culture samples

Two RDT products were selected for further testing: The SD Bioline RDT and the Creative Diagnostics RDT. A panel of reference and clinical strains was made including six *Salmonella* serovars and 13 other pathogens and contaminants commonly isolated from blood cultures or with possible cross-reactivity (Table 2). Freshly grown colonies were suspended in sterile saline at 0.5 McFarland (which equals 1,5 x 10^8^ CFU/ml). The estimated bacterial concentration after serial dilutions were performed was 375 CFU/ml. Then, 500 µl of this bacterial suspension was used to spike blood culture bottles (BacT/ALERT FA/PF), to which defibrinated sheep blood (ref. 8545104, International Medical Products, Marche-en-Famenne, Belgium) had been added. The blood culture bottles were incubated at 35°C in a BacT/ALERT® 3D 60 machine (bioMérieux, Marcy L’Etoile, France) until growth. Duration of incubation varied between 12–20 hours (S1 Table). Tests were performed on the same day. Colony counts were performed to estimate the concentration of bacteria present in the grown blood culture broth. The calculated concentrations varied between 10^7^−10^10^ CFU/ml depending on the organism (S1 Table). The manufacturers’ instructions were modified in accordance to the optimizations tests performed: 1.5 ml of grown blood culture broth was centrifuged for 1 min. at 500 x *g* in a 1.5-ml tube. A calibrated micropipette was used to transfer the supernatant to the RDT products. For the SD Bioline RDT, 100µl of supernatant was transferred. For the Creative Diagnostics RDT, application of 100µl of supernatant was compared to the application of 50 µl of supernatant with addition of 4 drops of provided buffer. Results of the SD Bioline RDT were read after 10 and 20 minutes as the IFU reported a reading window of 10–20 minutes. The result of the Creative Diagnostics RDT were read at 10 minutes as mentioned in the IFU.

**Table 2:** Bacterial strains used for spiking blood culture bottles and corresponding rapid diagnostic test results.

			RDT^a^	
			SD Bioline	Creative Diagnostics
Species/strain	Strain reference no.	Group O and H antigens	Target: *Salmonella* Typhi	Target: *Salmonella* spp.
*Salmonella enterica* subsp. *enterica*				
*Salmonella* Paratyphi A	ATCC 9150	A, 1, 2, 12;a [1,5]	Negative	Positive
*Salmonella* Typhimurium	ATCC 14028	B, 4, 5, 12; i, 1, 2	Negative	Positive
Salmonella Concord	*	C_1_, 6,7;l,v,1,2	Negative	Negative
*Salmonella* Typhi	00032304	D_1_, 9, 12 [Vi]; d	Positive	Positive
*Salmonella* Enteritidis	ATCC 13076	D_1_, 1, 9, 12:g, m	Positive	Positive
*Salmonella* Jamaica	*	D_1_, 9,12;r,1,5	Positive	Positive
*Salmonella enterica* subsp. diarizonae	*	1:i:z53	Negative	Negative
*Escherichia coli*	ATCC 25922		Negative	Negative
*Klebsiella pneumoniae*	ATCC 13883		Negative	Invalid
*Burkholderia cepacia*	*		Negative	Negative
*Acinetobacter baumannii*	*		Negative	Negative
*Citrobacter freundii* Vi+	*		Negative	Positive
*Proteus mirabilis*	ATCC 12453		Negative	Positive
*Enterobacter cloacae*	ATCC 23355		Negative	Negative
*Streptococcus pneumoniae*	ATCC 6305		Negative	Negative
*Staphyloccus aureus*	ATCC 25923		Negative	Negative
Coagulase negative staphylococci	*		Negative	Negative
*Bacillus cereus*	*		Negative	Negative
*Corynebactrium diphtheriae*	*		Negative	Negative

* = Confirmed strains from external quality controls or routine patient care

^a^ = Results after application of 100µl of blood culture supernatant

00032304 = strain confirmed at the Belgian Reference Laboratory for *Salmonella*, Scientific Institute of Health (WIV-ISP), Etterbeek, Belgium

ATCC = American Type Culture Collection; SD = Standard Diagnostics; RDT = Rapid Diagnostic Test

### III Application on stored grown blood culture broth

A selection was made out of a collection of blood culture broth samples previously collected and stored at -80°C at the Institut National de Recherche Biomédicale (INRB) in Kinshasa, DRC, and the Sihanouk Hospital Center of HOPE (SHCH) in Phnom Penh, Cambodia. Both institutes conduct microbiological surveillance in collaboration with the Institute of Tropical Medicine (ITM) in Antwerp, Belgium.

At SHCH, blood (2 x 10 ml) was cultured in BacT/ALERT culture bottles (paired aerobic (FA) and anaerobic bottles (FAN), bioMérieux, Marcy l’Etoile, France). Blood cultures were worked up as previously described [14]. The majority of samples were tested on site, a selection of samples were tested at ITM, Antwerp, Belgium, after shipment on dry ice. In DRC, 1–4 mL of blood was sampled into pediatric blood culture bottles (BacT/ALERT PF) for children (≤ 14 years old), and 2 × 10 mL into aerobic adult blood culture bottles (BacT/ALERT FA). Blood cultures were processed as described elsewhere [15]. All samples from DRC were shipped to Belgium on dry ice for testing.

The blood culture broth samples comprised three groups: (i) *Salmonella* positive samples; (ii) samples positive for competing organisms; and (iii) non-grown blood cultures. All tests were performed between August 2016 and January 2017. For this step, 1.5 ml of grown blood culture broth was centrifuged for 1 min. at 500 x *g* in a 1.5-ml tube and 100µl supernatant was applied to both RDT products. All results were read by two readers. One of the readers had access to the blood culture test results, but sample numbers were coded before testing. The second reader had no access to the blood culture results and the two readers were blinded to each other. In case of discordance, the final result was based on consensus.

In case of false positive or false negative RDT results, the blood culture broth was plated onto sheep blood agar and incubated overnight and next re-identified by standard biochemical methods. In case the re-identification was different from the identification recorded in the database, the sample was excluded from subsequent analysis.

### Evaluation of instructions for use (IFU) and readability of test lines

The information mentioned in the IFU of the RDT products was assessed using a checklist that was based on a generic template for IFUs for malaria RDTs from a previous study [16]. In addition, cassette design and the ease of reading test lines was assessed.

### Sample size calculation

To achieve an estimated sensitivity of 95% with an accuracy of 0.05, 73 samples containing *Salmonella* spp. (target Creative Diagnostics RDT) and 73 samples containing *Salmonella* Typhi (target SD Bioline RDT) were needed. To achieve an estimated specificity of 90% with an accuracy of 0.05, 139 controls, meaning samples other than those positive for *Salmonella* spp. and/or *Salmonella* Typhi were needed. The control group included samples with: (i) other *Salmonella* serotypes, (ii) isolates belonging to the most frequently isolated pathogens and contaminants at the respective study sites in Cambodia and the DRC (iii) isolates of species with potential cross-reactivity.

### Statistical analysis

Sensitivity and specificity of the RDT products compared to the standard test (blood culture) were calculated with 95% confidence intervals (CI) using Stata 12 (Stata Corp., College Station, TX, USA). Inter-observer agreements for test line intensities and positive and negative test results were expressed by the percentage of overall agreement and by kappa values for each pair of readers. Reader one was the same lab technician for all samples, while the second reader was either a lab technician based in Cambodia (‘reader 2’) or in Belgium (‘reader 3’).

### Ethical review

The microbiological surveillance study through which blood culture broth was sampled and stored was approved by the Institutional Review Board (IRB) of ITM, the Ethical Committee of Antwerp University, Belgium, the National Ethics Committee for Health Research (NECHR), Cambodia and the ethics committee of the ‘École de Santé Publique’ of the University of Kinshasa, DRC. In addition, specific approval was requested for the use of these stored blood culture samples for the evaluation of antigen-based *Salmonella* rapid diagnostic tests and which was granted by the IRB of ITM (ref. 629/08, dated 02/07/2014), the NECHR in Cambodia (ref. 0359 NECHR, dated 29/12/2014) and the ethics committee of the ‘École de Santé Publique’ of the University of Kinshasa, DRC (ref. ESP/CE/072/2015, dated 07/07/2015). No consent was asked from patients as it concerned coded left-over samples collected through routine care.

## Results

### I Proof-of-concept evaluation with determination of limit of detection

To determine the LOD for each product and its intended target (s), a *Salmonella* dilution series (range 1.5 x 10^8^ CFU/ml to 1.5 x 10^3^ CFU/ml) was applied to each RDT product. The results are shown in Table 3. In the IFU of three out of six RDT products, no information was mentioned about analytical sensitivity. For the SD Bioline RDT a LOD of 10^5^ CFU/ml for *Salmonella* Typhi was mentioned and for Creative Diagnostics this was 10^7^ CFU/ml for *Salmonella* Typhi and 10^4^ CFU/ml for *Salmonella* Enteritidis and *Salmonella* Typhimurium. For the Dialab RDT an analytical sensitivity of 25 ng/ml LPS for *Salmonella* Typhi and Paratyphi A was mentioned without stating a conversion factor to CFU/ml.

**Table 3:** Determination of limit of detection and corresponding test line intensity for each rapid diagnostic test.

		LOD for *Salmonella* Typhi (CFU/ml)		LOD for *Salmonella* Paratyphi A (CFU/ml)		LOD for *Salmonella* Typhimurium (CFU/ml)		LOD for *Salmonella* Enteritidis (CFU/ml)	
	Dilution medium	PBS	Broth	PBS	Broth	PBS	Broth	PBS	Broth
Product name	Test line								
One Step *Salmonella* Typhi Ag Rapid Detection Kit	*Salmonella* Typhi	10^7^/10^8^ (W)	10^7^/10^8^ (W)	-	-	-	-	-	-
*Salmonella* Ag Rapid Test	*Salmonella* spp.	10^8^ (F)	10^8^ (F)	10^8^ (F)	10^8^ (F)	10^8^ (W)	10^8^ (F)	10^7^ (F)	10^7^ (F)
"DIAQUICK" S. typhi/paratyphi Ag cassette	*Salmonella* Typhi	>10^8^	>10^8^	-	-	-	-	-	-
	*Salmonella* Paratyphi A	-	-	>10^8^	>10^8^	-	-	-	-
Accucare *S*. Typhi-*S*. Paratyphi Direct Antigen Detection kit	*Salmonella* Typhi	>10^8^*	10^7^ (W/F)	-	-	-	-	-	-
	*Salmonella* Paratyphi A	-	-	>10^8^*	>10^8^*	-	-	-	-
*Salmonella* Typhi Antigen Strip	*Salmonella* Typhi	>10^8^	>10^8^	-	-	-	-	-	-
*Salmonella* typhi/paratyphi A, B & C Test Device	*Salmonella* Typhi	>10^8^/10^8^ (F)	10^4^ (F)	-	-	-	-	-	-
	*Salmonella* Paratyphi A	-	-	>10^8^	>10^8^	-	-	-	-

Test line intensities are mentioned between brackets

>10^8^ = No test line visible at the highest concentration tested (10^8^ CFU/ml)

* = Faint test lines were visible at random concentrations, but at the highest concentration (10^8^ CFU/ml) no test line was visible

/ = In case of different results between parallel testing both results are mentioned with a "/" in between

CFU = Colony Forming Units; F = faint; LOD = Limit Of Detection; M = medium; PBS = Phosphate Buffered Saline; S = strong; W = weak

The SD Bioline RDT (detection of *Salmonella* Typhi) performed best with a LOD for *Salmonella* Typhi of 10^7^−10^8^ CFU/ml, no invalid test results and no false positive results, for both PBS and blood culture broth. The Creative Diagnostics RDT also performed satisfactory with an LOD of 10^7^ CFU/ml for *Salmonella* Enteritidis and 10^8^ CFU/ml for *Salmonella* Typhi, *Salmonella* Paratyphi A and *Salmonella* Typhimurium. The other tests performed unsatisfactory (no test line visible at 10^8^ CFU/ml) and we therefore proceeded the analysis with the SD Bioline RDT and the Creative Diagnostics RDT.

### II Application on spiked blood culture samples

Nineteen blood culture bottles were spiked with a panel of reference and clinical strains including six *Salmonella* serovars and 13 other pathogens (Table 2). All bacteria grew in the blood culture bottles within 24 hours.

For the SD Bioline RDT (targeting *Salmonella* Typhi) the blood culture broth samples containing *Salmonella* Typhi, *Salmonella* Enteritidis and *Salmonella enterica* serovar Jamaica (all serogroup D) tested positive. The remaining blood culture samples containing *Salmonella* serotypes other than serogroup D or competing organisms tested negative. There were no invalid test results.

The Creative Diagnostics RDT showed a visible test line for all *Salmonella* serovars except *Salmonella* Concord (serogroup C1) and *Salmonella enterica* subspecies diarizonae. Application of 100 µl of supernatant and no buffer (results shown in Table 2) resulted in two samples with a false positive reading, those containing *Proteus* spp. and *Citrobacter freundii*. One invalid result occurred (sample containing *Klebsiella pneumoniae*) due to poor background clearing. Addition of buffer resulted in better background clearance but also in more false-positive readings and was therefore discontinued.

### III Application on stored grown blood culture broths

In total 413 clinical blood culture broth samples from 413 patients were thawed and subsequently applied to both RDT products (Table 4; S2 Table). Median storage time at -80°C before testing was 23 months (interquartile range (IQR) 18–25 months). The median age of patients with a *Salmonella* infection was 4 years (IQR 1–25 years) and 54.9% (152/277) were male; the median age of the patients without a *Salmonella* infection was 29 years (IQR 2–60 years) and 46.2% (61/132) were male. Thirteen (3.1%) samples were excluded from subsequent analysis as they gave false negative or false positive results with growth of a pathogen different from the one recorded in the database upon subculture of the thawed blood culture broth (S3 Table).

**Table 4:** Results of the SD Bioline and Creative Diagnostics rapid diagnostic tests on blood culture broth.

	No. of samples from Cambodia	No. of samples from DRC	Total no. of samples	No. SD Bioline	No. Creative Diagnostics
Stored blood culture broths tested				positive (%)	positive (%)
*Salmonella* panel					
*Salmonella* Typhi	36	37	73	73 (100)	70 (95.9)
*Salmonella* Enteritidis	1	67	68	65 (95.6)	67 (98.5)
*Salmonella* Paratyphi A	62	0	62	0	9 (14.5)
*Salmonella* Typhimurium^a^	0	70	70	2 (2.9)	70 (100)
*Salmonella* Choleraesuis	4	0	4	0	1 (25.0)
Competing panel					
Gram-negative organisms					
*Escherichia coli*	8	12	20	0	2 (10.5)*
*Klebsiella pneumoniae*	7	8	15	0	2 (14.3)*
*Pseudomonas* species^b^	5	3	8	0	1 (12.5)
*Acinetobacter* species^c^	3	4	7	0	1 (14.3)
*Enterobacter* species^d^	6	4	10	0	1 (10.0)
*Burkholderia pseudomallei*	4	0	4	0	0
*Burkholderia cepacia*	1	0	1	0	0
*Serratia species*	0	2	2	0	0
*Aeromonas* species	1	0	1	0	0
*Moraxella* species	1	0	1	0	0
Gram-positive organisms					
*Staphylococcus aureus*	4	8	12	0	1 (8.3)
*Streptococcu*s species^e^	7	0	7	0	0
*Enterococcus* species^f^	2	4	6	1 (16.7)	0
*Bacillus* species	5	4	9	0	0
*Staphylococcus non-aureus*	7	10	17	1 (5.9)	3 (17.6)
*Corynebacterium* species	1	0	1	0	0
*Candida* species^g^	0	2	2	0	1 (50.0)
No growth^h^	8	5	13	0	0

^a^ = Including *Salmonella enterica* serovar Typhimurium var. Copenhagen (n = 3)

^b^ = Including *Pseudomonas aeruginosa* (n = 2), *Pseudomonas stutzeri* (n = 2), *Pseudomonas* species (n = 3), *Pseudomonas alcaligenes* (n = 1)

^c^ = Including *Acinetobacter baumannii* (n = 2 *Acinetobacter lwoffii* (n = 1), *Acinetobacter* species (n = 5)

^d^ = Including *Enterobacter cloacae* (n = 3), *Enterobacter agglomerans* n = 1), *Enterobacter* species (n = 6)

^e^ = Including *Streptococcus pneumoniae* (n = 3), *Streptococcus* group A (GAS) (n = 2), *Viridans streptococci* (n = 2)

^f^ = Including *Enterococcus faecalis* (n = 1), *Enterococcus* species (n = 3), *Enterococcus faecium* (n = 2)

^g^ = Including *Candida tropicalis* (n = 1) and *Candida* species (n = 1)

^h^ = After 7 days of incubation

* = 1 invalid result

DRC = Democratic Republic of the Congo; SD = Standard Diagnostics

The 413 broth samples included for the final analysis comprised 240 samples from DRC and 173 from Cambodia; 277 samples had been tested positive with *Salmonella*; 123 had grown other competing bacteria and 13 were samples without growth (Table 4).

Invalid test results were observed only twice, both for Creative Diagnostics. These invalid results were due to very poor background clearance. One SD Bioline blister contained a discolored (green) desiccant (indicating humidity saturation) and was subsequently replaced by another cassette.

The SD Bioline RDT correctly identified *Salmonella* Typhi antigen in 73/73 (100%) blood culture samples, corresponding to a sensitivity of 100% (95%-CI [95.1–100%]). The specificity was 79.7% (95%-CI [75–83.9%]) with 69/340 (20.3%) of the non-*Salmonella* Typhi samples giving positive RDT results. These samples contained: *Salmonella* Enteritidis (n = 65), *Salmonella* Typhimurium (n = 2), *Staphylococcus* non-*aureus* (n = 1) and *Enterococcus faecium* (n = 1).The sensitivity and specificity of the SD Bioline for the detection of *Salmonella* Typhi and *Salmonella* Enteritidis combined (both serogroup D *Salmonella*) were 97.9% (95%-CI [93.9–99.6%]) and 98.5% (95%-CI [96.3–99.6%]).

True positive test results (defined here as positive for group D *Salmonella*) showed strong test line intensities in 105/138 (76.1%), medium in 19/138 (15.9%), weak in 9/138 (6.5%) and faint in 5/138 (3.6%) of cases. Of the four false positive results, one gave a strong test line (*Salmonella* Typhimurium), one medium and two faint. One test result (*Salmonella* Typhi) turned positive only at 20 minutes, while all other true positive tests results were visible at 10 minutes.

The Creative Diagnostics RDT correctly identified 217/277 (78.3%) *Salmonella* positive blood culture samples corresponding to a sensitivity of 78.3% (95%-CI [73–83%]). False negative results were caused by samples containing *Salmonella* Paratyphi A (85.5% (53/62) of all *Salmonella* Paratyphi A positive samples tested negative), *Salmonella enterica* serovar Choleraesuis (*Salmonella* Choleraesuis) (3/4; 75% tested negative), *Salmonella* Typhi (3/73; 4.1%) and *Salmonella* Enteritidis (1/68; 1.5%). Of note, the three *Salmonella* Typhi positive samples that gave no visible test line for Creative Diagnostics did give a clearly visible test line with the SD Bioline RDT.

Specificity was 91% (95%-CI [84.9–95.3%]) with 12/134 samples containing no *Salmonella* giving false positive results. These samples included *Escherichia coli* (n = 2), *Klebsiella pneumoniae* (n = 2), *Pseudomonas* species (n = 1), *Acinetobacter baumannii* (n = 1), *Enterobacter cloacae* (n = 1), *Staphylococcus* non-*aureus* (n = 3), *Staphylococcus aureus* (n = 1) and *Candida tropicalis* (n = 1).

When considering only *Salmonella* Typhi, *Salmonella* Enteritidis and *Salmonella* Typhimurium as true positive results, Creative Diagnostics correctly identified 207 out of 211 (98.1%) positive samples resulting in a higher sensitivity of 98.1% (95%-CI [95.2–99.5%]).

In case of a true positive result, the Creative Diagnostics RDT showed strong test line intensities in 6/217 (2.8%), medium in 61/217 (28.1%), weak in 128/217 (59%) and faint in 22/217 (10.1%) of cases. Of the 12 false positive results, one gave a strong test line (*Staphylococcus* non *aureus*), and 11 faint.

**Fig 1:**
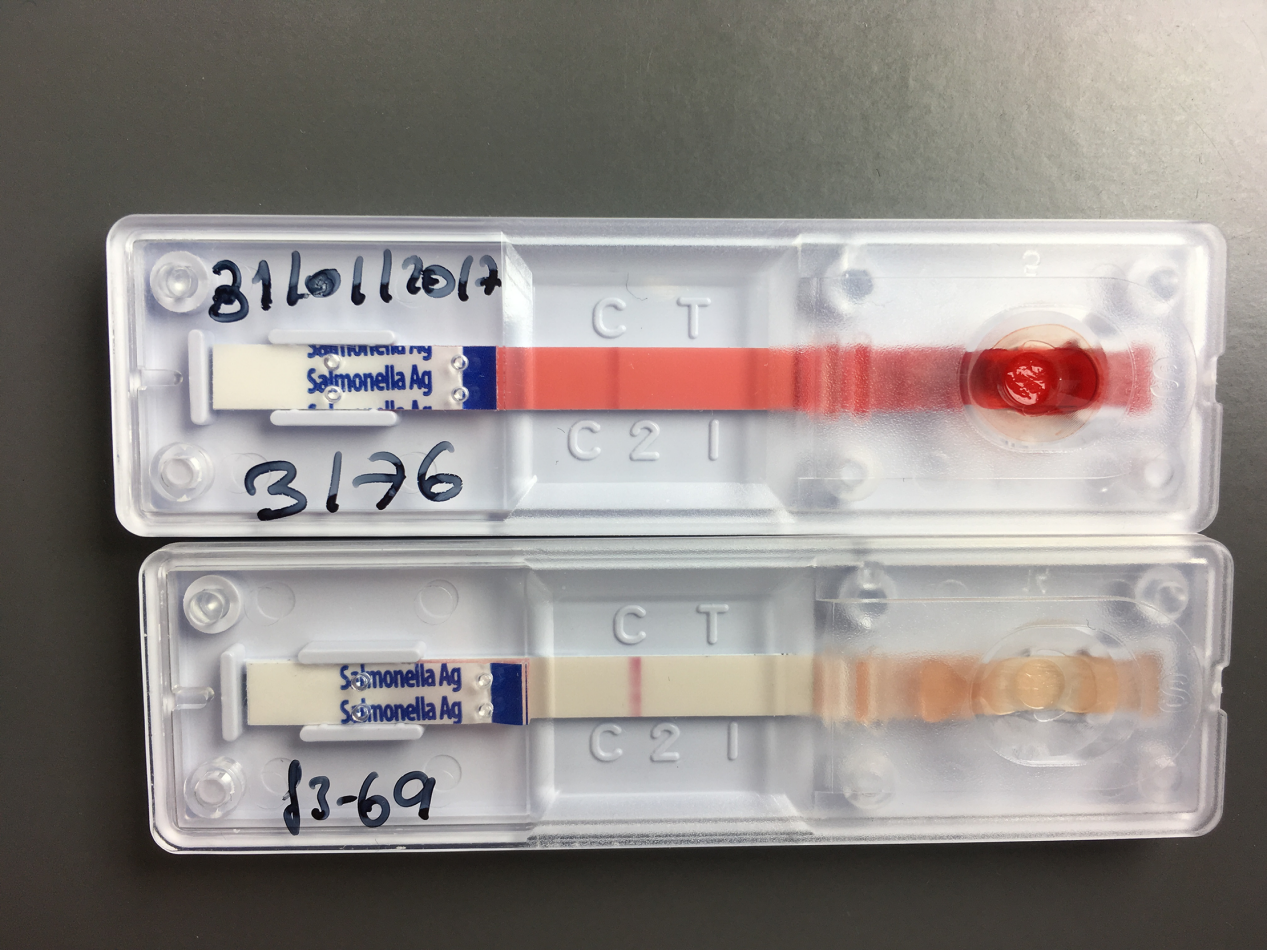
Variable background clearance. Both tests shown (Standard Diagnostics Bioline One Step *Salmonella* Typhi Ag Rapid Detection Kit) with the presence of a control line and absence of a test line, show a negative test result. Background clearance is scored poor for the test above (more reddish) and good for the test below.

The degree of background clearance was more associated with the geographical origin of the samples than with the RDT products. Samples from Cambodia generally had a poorer background clearance compared to samples from DRC (Fig 1). We hypothesize that this might be caused by (inadvertent) repeat freeze-thawing caused by occasional power failure at the DRC site. Reading of results in case of poor background clearance was more difficult for the Creative Diagnostics RDT, probably due to the weaker intensity of control- and test lines.

**Fig 2:**
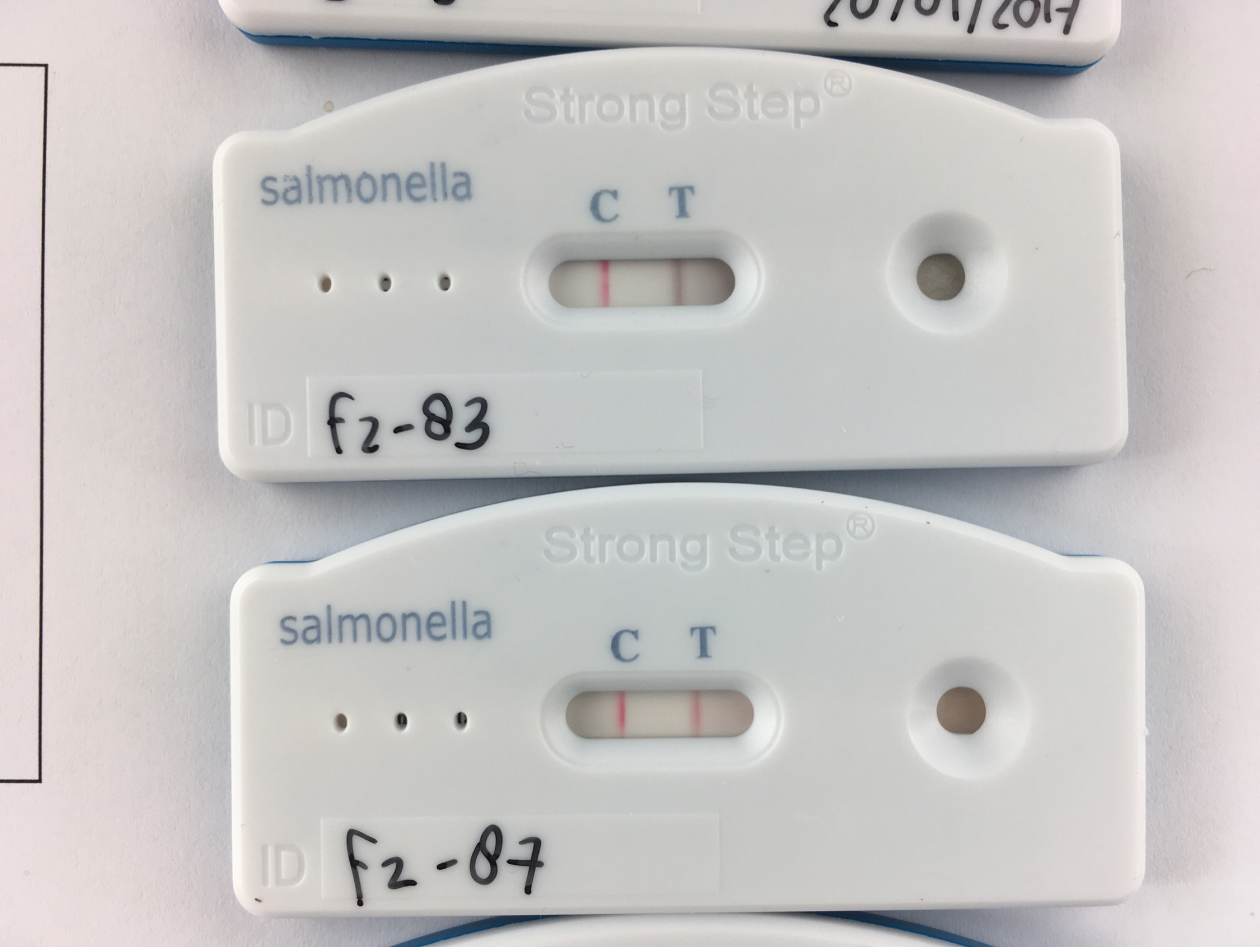
Presence of black and red colored test lines. Samples applied to both tests (*Salmonella* Ag Rapid Test, Creative diagnostics) contained *Salmonella* Enteritidis and both were scored as a positive test result. The test above shows a red colored control line and a black colored test line. The picture below shows a red colored control and test line.

Of note, occurrence of faint black test lines or combined red and black lines were occasionally observed (Fig 2). The IFU’s of both products did not mention a possible presence of black colored test lines or combined black and red colored test lines. For the current evaluation, faint black test lines were reported as negative; black test lines that were scored as weak, medium or strong and test lines with a combined red and black color were reported as positive. Occurrence of black or combined red and black lines were mostly seen for samples from DRC and for samples containing *Salmonella* Typhimurium.

For the SD Bioline RDT, overall agreement and kappa values between pairs of observers were excellent for both positive and negative readings (≥ 97.9%, kappa values ≥0.95) and for line intensity readings (≥ 96.1%, kappa values ≥ 0.93) (S4 Table). All but one discordance in line intensity occurred within one category of difference. For Creative Diagnostics, agreement was good for positive and negative readings (≥ 96.2%, kappa values ≥ 0.92) and moderate for line intensity readings (≥ 86.0%, kappa values ≥ 0.80) (S4 Table). All but two discordances in line intensity occurred within one category of difference.

### Evaluation of instructions for use (IFU) and readability of test lines

The information mentioned in the IFU of all 6 RDT products was assessed (S5 Table); the most striking findings are mentioned here. All of the 6 RDT kits contained an IFU of which one had an additional job aid (SD Bioline). Version numbers and dates of issue were both missing in 3 out of 6 IFUs. Only one IFU clearly mentioned the target antigen (Dialab RDT). Except for the SD Bioline RDT kit, detailed information on the performance of the RDT products was lacking. Three IFUs did not provide any information on sensitivity or specificity; two IFUs gave vague indications of sensitivity and specificity such as ′Sensitivity: ACCUCARE S.typhi-S. paratyphi assay was run using serum and stool samples versus culture positive samples and found to give positive results in all cases‘ (Labcare RDT). Some IFUs contained major errors, like the SMI Typhi RDT IFU which mentioned that it detected *Salmonella* Typhimurium instead of *Salmonella* Typhi. Of note, the Creative Diagnostics IFU mentioned a green colored test line while we observed a red colored test line for both the control and test line.

For reading of results, SD Bioline was preferred compared to Creative Diagnostics. Control- and test lines had a stronger intensity, and test lines remained visible for more than 24 hours. For the Creative Diagnostics RDT, positive test lines were often faint to weak and the visibility of the lines was heavily influenced by variable background clearance.

## Discussion

In this study, the performance of immunochromatographic rapid diagnostics tests for the detection of *Salmonella* antigens in blood culture broth was evaluated.

The SD Bioline RDT showed a sensitivity of 100% which is in line with the 96.7% previously observed for this product in Laos [8]. The specificity of 80.2% was lower due to the inclusion of a high number of *Salmonella* Enteritidis positive samples. The positive results for samples containing serogroup D *Salmonella* adds evidence to the assumption of Castonguay *et al*., that this product probably detects the O9 antigen [8]. The specificity of the SD Bioline RDT for the detection of group D *Salmonella* combined (98.9%) was much better compared to *Salmonella* Typhi alone. Two out of three false-positive results occurred with samples containing probable contaminants and it cannot be excluded that group D *Salmonella* might have been present in the blood at the time of sampling.

The LOD of the SD Bioline was determined to be 10^7^−10^8^ CFU/ml for *Salmonella* Typhi which was lower than mentioned in the IFU (10^5^ CFU/ml) but which is in line with the estimated minimal bacterial concentrations required for blood cultures to be flagged positive 10^7^−10^8^ CFU/ml) [17].

The Creative Diagnostics RDT had not been evaluated previously. In the present study, a sensitivity and specificity of 78.3% and 91.0% were found. The highest proportion of false negative results were observed for samples containing *Salmonella* Paratyphi A. A possible explanation might be that the test detects the O12 antigen. This antigen, which can be subdivided into subtypes 12_1_, 12_2_, and 12_3_, is present in *Salmonella* Typhi, Typhimurium and Enteritidis and absent in *Salmonella* Choleraesuis and *Salmonella* Concord. *Salmonella* Paratyphi A carries only subtypes 12_1_ and 12_3_ [18]. Tam *et al*. noted that the O12 antibody is less immuno-dominant than O9 (or O2) and that although most typhoid patients produce vast amounts of anti-O12 antibodies, many paratyphoid patients produce little or none at all [19]. In addition, PCR experiments showed that the median number of copies of target DNA per ml of blood was 39 for *Salmonella* Paratyphi A compared to 60 for *Salmonella* Typhi which suggests a lower concentration in blood during infection [20, 21].

The LOD was determined to be 10^8^ CFU/ml for *Salmonella* Typhi, Paratyphi A and Typhimurium and 10^7^ CFU/ml for *Salmonella* Enteritidis. Similar to the SD Bioline RDT, these LODs are lower compared to what is mentioned in the IFU for detection in stool. Of note, three *Salmonella* Typhi positive samples that gave no visible test line with the Creative Diagnostics RDT, did give a clearly visible test line with the SD Bioline which could indicate a slightly lower LOD of the Creative Diagnostics RDT in practice. The 12 false positive results were caused by samples confirmed to contain a range of clinically significant pathogens such as *Escherichia coli* and *Klebsiella pneumoniae* and is thus of concern.

The remaining four RDT products did not reliably detect *Salmonella* in the highest concentration applied (10^8^ CFU/ml) in PBS and blood culture medium. This raises questions regarding the accuracy of these RDT products on blood samples (serum, plasma, whole blood), as the concentration of *Salmonella* Typhi and *Salmonella* Paratyphi A in blood during acute infection is estimated to be only 1 CFU/ml [5, 22]. In addition, the quality of information provided in the IFU’s of these products was generally poor.

The present study has some limitations. First, clinical blood culture broth samples were tested retrospectively after storage times up to 7 years. Second, freezing and thawing of samples might have negatively influenced the test results due to deterioration of the samples. Last, it was not possible to reconfirm the identification of all blood culture broth samples that gave false results, either because bacteria could not be recovered from samples or because of assumed overgrowth by contaminants.

Apart from these limitations, this study also has several strengths. We assessed the use of *Salmonella* RDT products for the detection of several *Salmonella* serotypes, which is relevant since the epidemiology of invasive *Salmonella* infections is changing globally. In addition, the possible cross- reactivity of a range of competing pathogens was assessed. Last, samples from two geographically distinct settings, using different blood culture bottles, were used. Both settings are endemic for invasive *Salmonella* infections.

The use of an RDT on grown blood culture broth has the advantage of speeding up the diagnosis of invasive bloodstream infections, or even improving detections rates [8, 23, 24]. However, in Asia the addition of the SD Bioline RDT to the routine work-up of blood culture samples would likely not be of major benefit. Despite its overall good performance, the product is unable to detect *Salmonella* Paratyphi A which in some Asian countries now causes more infections than *Salmonella* Typhi [25]. The product would also not be suited for use in sub-Saharan Africa as it is not able to detect *Salmonella* Typhimurium which is commonly isolated from blood cultures in this setting [15].

The Creative Diagnostics RDT might be of use in sub-Saharan Africa as it was able to detect nearly all samples positive with *Salmonella* Typhi, Enteritidis and Typhimurium, the serotypes that cause nearly all *Salmonella* bloodstream infections in this setting [15]. The bad performance with *Salmonella* Paratyphi A and *Salmonella* Choleraesuis positive samples limits its use in Asia. In addition, the operational characteristics of Creative Diagnostics RDT (line intensities, line stability) need to be improved.

In general, the need for grown blood culture broth requires the presence of an adequately equipped laboratory. Although the time to diagnosis can be shortened with 1–2 days future studies should evaluate if point of care testing is possible. Of concern is the fact that a percentage of negative samples gave black test lines, which might be attributed to the presence of charcoal in the culture media of the blood culture bottles used in this study. Further validation with other types of blood culture bottles is thus needed. Although easily distinguishable from true positive test lines by trained readers, misinterpretation in the field is likely.

The present study shows that new RDT products for the detection of invasive *Salmonella* infections need to be developed, in particular for the reliable detection of *Salmonella* Paratyphi A. Operational characteristics, instructions for use and specificity of existing products should be further optimized.

## Conclusion

The SD Bioline RDT had an excellent sensitivity and specificity for the detection of *Salmonella* Typhi and *Salmonella* Enteritidis in grown blood culture broth. The Creative Diagnostics RDT had a moderate sensitivity for the detection of *Salmonella* spp. and an acceptable specificity; it particularly failed to detect *Salmonella* Paratyphi A.

## Supporting information

S1 Checklist STARD Checklist. (PDF)

S1 Table Dataset—Results spiking experiments. ATCC = American Type Culture Collection.
CFU = Colony Forming Units.
NA = Not Applicable.
(XLSX)

S2 Table Dataset—Results Rapid Diagnostic tests on blood culture broth. N = no test line; F = faint; W = weak; M = medium; S = strong.
Min = Reading after 10 minutes.
Max = Reading after 20 minutes.
R1 = Reader 1.
R2 = Reader 2 (2 different persons).
(XLSX)

S3 Table Dataset–Blood culture broth samples excluded from analysis. Min = Reading after 10 minutes.
Max = Reading after 20 minutes.
R1 = Reader 1.
R2 = Reader 2 (2 different persons).
(XLSX)

S4 Table Results Rapid Diagnostic tests on blood culture broth—Kappa values. Ag = Antigen.
SD = Standard Diagnostics.
R1 = reader one (lab technician—first reader in both Belgium and Cambodia).
R2 = reader 2 (lab technician—second reader in Cambodia).
R3 = reader 3 (lab technician—second reader Belgium).
(XLSX)

S5 Table Analysis Instructions For Use (IFU). IFU = Instructions For Use.
RDT = Rapid Diagnostic Test.
(XLSX)
